# Identifying treatment-responsive patient subgroups in a neutral clinical trial of Intensive blood pressure reduction in acute intracerebral hemorrhage: A post hoc explainable machine learning analysis

**DOI:** 10.1016/j.neurot.2026.e00972

**Published:** 2026-07-23

**Authors:** Anh T. Tran, Adnan I. Qureshi, Junhao Wen, Joshua Z. Willey, David Roh, Santosh B. Murthy, Mert R. Sabuncu, Ajay Malhotra, Jennifer A. Kim, Guido J. Falcone, Lee H. Schwamm, Kevin N. Sheth, Seyedmehdi Payabvash

**Affiliations:** a Department of Radiology, Columbia University Irving Medical Center, New York, NY, 10032, USA; b Zeenat Qureshi Stroke Institute and Department of Neurology, University of Missouri, Columbia, MO, 65212, USA; c Department of Neurology, Columbia University Irving Medical Center, New York, NY, 10032, USA; d Department of Neurology, Weill Cornell Medical College, Cornell University, New York, NY, 1006, USA; e Department of Radiology, Weill Cornell Medical College, New York, NY, 10065, USA; f School of Electrical and Computer Engineering, Cornell Tech, Cornell University, New York, NY, 10044, USA; g Department of Radiology and Biomedical Imaging, Yale School of Medicine, New Haven, CT, 06520, USA; h Department of Neurology, Yale School of Medicine, New Haven, CT, 06520, USA

**Keywords:** Intracerebral hemorrhage, Blood pressure, Explainable machine learning, Counterfactual

## Abstract

The Antihypertensive Treatment of Acute Cerebral Hemorrhage (ATACH-2) trial reported no overall benefit from intensive blood pressure (BP) reduction in intracerebral hemorrhage (ICH), potentially masking benefit in select patient subgroups. We evaluated whether explainable machine learning could identify treatment-arm subgroups in whom BP reduction is associated with improved outcomes. Using the ATACH-2 dataset, we trained an XGBoost model on two-thirds of the control arm (n = 326) to predict 3-month poor outcome (modified Rankin Scale >3). The model was then applied to treatment-arm patients (n = 499) and held-out controls (n = 163). SHapley Additive exPlanations (SHAP) quantified individual BP risk contributions, and counterfactual perturbation simulated BP reductions. We applied an exploratory, hypothesis-generating grid search to identify selection strategies yielding the lowest odds ratio (OR) for poor outcome in treatment subgroups versus controls. The model achieved an area under the curve of 0.85 (95% confidence interval [CI], 0.81–0.89) in cross-validation and 0.83 (95% CI, 0.77–0.89) in independent validation. Using a combined SHAP and counterfactual analysis, we identified a candidate treatment-responsive subgroup (n = 56) in whom intensive BP reduction was independently associated with lower odds of poor outcome compared with held-out controls (OR = 0.21, 95% CI, 0.08–0.54; p = 0.002). This association remained significant when compared with the subset of held-out controls meeting the same selection criteria. Compared with the remainder of their respective groups, these patients had higher baseline systolic BP, more severe neurological deficits, lower blood glucose levels, and more frequent basal ganglia involvement. These findings highlight the potential of explainable machine learning for precision BP management in acute ICH.

## Introduction

Intracerebral hemorrhage (ICH) is the most disabling form of stroke [1]. In acute ICH patients, elevated blood pressure (BP) is a predictor of hematoma expansion and poor outcomes [2,3], making BP reduction a biologically compelling treatment target. Nevertheless, early randomized trials such as the second Intensive Blood Pressure Reduction in Acute Cerebral Hemorrhage (INTERACT2) [4] and Antihypertensive Treatment of Acute Cerebral Hemorrhage (ATACH-2) [5] had overall equivocal results. More recently, however, accumulating evidence from INTERACT3 [6] and INTERACT4 [7] trials, observational studies [8], and post-hoc analysis of prior trials [[9], [10], [11]] supports benefit of antihypertensive therapy, highlighting the importance of ultra-early (within 2 hours) intensive and sustained BP reduction in improving acute ICH outcomes. These findings suggest that uniform BP-lowering strategies may fail to account for patients’ heterogeneity and obscure treatment benefits in subgroups most likely to benefit. Baseline neurological deficit, hematoma volume and location, and comorbidities likely interact to determine whether a given patient benefits from intensive BP lowering; yet conventional trial designs and statistical analyses may not adequately capture this complexity.

**Fig. 1:**
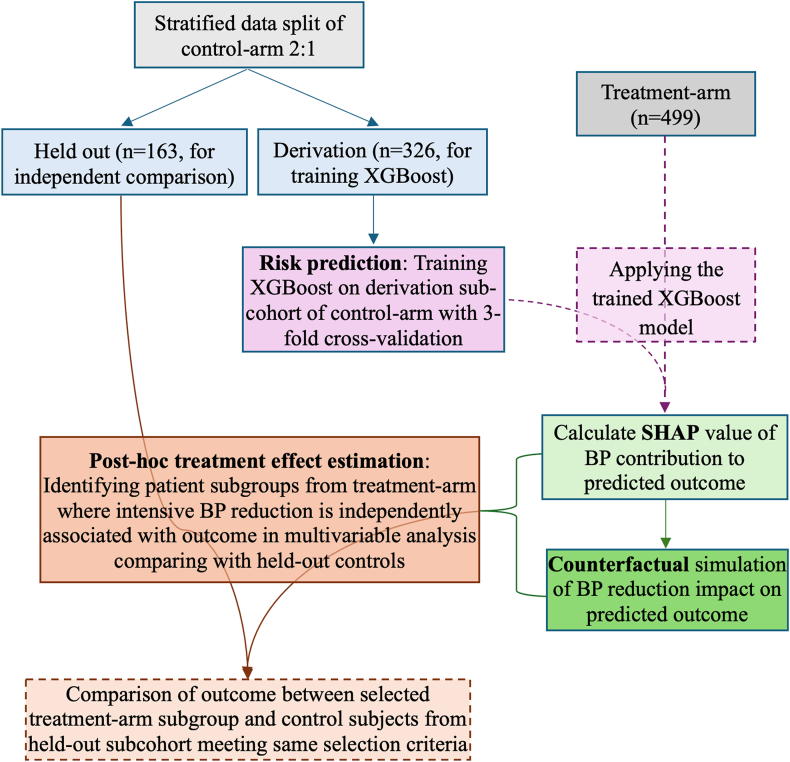
Analysis overview.

Explainable machine learning methods are well suited for modeling complex interactions among multiple risk factors by integrating high-dimensional clinical, laboratory, and imaging data while providing interpretable, patient-level insights into the determinants of predicted outcomes [12,13]. In this study, we applied two complementary explainability strategies to identify subgroups potentially responsive to intensive BP reduction among acute ICH patients in the treatment-arm of ATACH-2 trial, despite its overall neutral results (Fig. 1). Our analysis included two components: prediction of baseline outcome risk and estimation of treatment effects within selected subgroups. First, we trained a machine learning model using control-arm patients to estimate the probability of poor outcome under standard management, assuming that relationships learned in the control arm generalize to the broader study population. Next, we applied SHapley Additive exPlanations (SHAP) [12] to quantify the contribution of systolic and diastolic BP to individual-level predicted poor outcome risk. We also performed counterfactual simulations of BP reduction to identify patients in whom lowering BP is associated with a reduction in predicted risk. These explainability strategies generate hypotheses regarding potential treatment responsiveness. Finally, treatment effects were estimated by comparing outcomes between selected treatment-arm patients and held-out controls using multivariable regression. Overall, this post-hoc analysis explores the feasibility of using explainable machine learning to identify subgroups potentially responsive to treatment within an otherwise neutral trial.

## Methods

*Ethical approval*: The study was conducted in compliance with the Health Insurance Portability and Accountability Act (HIPAA) and approved by the Institutional Review Boards of Columbia University Medical Center and clinical trial participating sites with signed informed consent from study participants (AAAV3664).

*Patients*: We analyzed the ATACH-2 clinical trial dataset – a phase III study of intensive BP reduction in adults (>18 years old) with acute supratentorial ICH, smaller than 60 mL, who had at least one episode of systolic BP > 180 mmHg at presentation, and were enrolled across 110 sites in 6 countries [5]. The intervention was aimed to start within 4.5 hours of onset and reduce systolic BP to 110–139 mmHg but found no overall treatment benefit [5]. Poor outcome was defined by 3-month modified Rankin Scale (mRS) > 3. For patients without a recorded 90-day mRS, we used the mRS obtained at >90 days (n = 29), between 30 and 90 days (n = 11), or at 30 days (n = 21), in that order of availability.

*Training a baseline machine learning model to predict* 3-month *outcomes in acute ICH:* To train an outcome prediction model independent of any intensive BP reduction effects, we used patients from the standard BP management (control)-arm of the ATACH-2 trial (Fig. 1). Using outcome-stratified sampling and preserving event rates, control-arm patients were randomly divided (2-to-1) to a “training cohort” for the machine learning model and a “held-out cohort” for comparison with treatment-arm patient subgroups likely to benefit (Supplementary Table 1).

Algorithm

**Table:** 

Input:
Dataset $D={\left\{\left(x_{i},T_{i},Y_{i}\right)\right\}}_{i=1}^{N}$
Where:
$x_{i}\in R\;\left(baseline\;variables\right)$
$T_{i}\in \left\{0,1\right\}\;\left(1=treated,0=control\right)$
$Y_{i}\in \left\{0,1\right\}\;\left(binary\;poor\;outcome=1,favorable\;outcome=0\right)$

$$Let\;D_{c}=\{i:T_{i}=0\}\;\text{and}\;D_{T}=\{i:T_{i}=1\}\text{.}$$

Split $D_{c}$ into “derivation cohort” (train) versus “independent reference cohort” (test)

$$D_{c}=D_{c}^{\left(f,train/validation\right)}\cup D_{c}^{\left(f,test\right)}$$

We used XGBoost (eXtreme Gradient Boosting) as the supervised machine learning algorithm for outcome prediction. Supplementary Table 2 lists the variables used for outcome prediction by the model. XGBoost is an ensemble tree-based prediction algorithm that inherently captures nonlinear relationships and higher-order feature interactions, making it suitable for complex clinical data with heterogeneous predictors. The algorithm accommodates both continuous and categorical variables and natively handles missing data without any need for imputation. Notably, XGBoost supports the imposition of monotonicity constraints, which were applied to systolic and diastolic BP variables to ensure that predicted outcome risk changed in physiologically plausible direction. We used 3-fold cross-validation for hyperparameter optimization, followed by training on the full training subcohort using an XGBoost classifier base model with n_estimators = 300, max_depth = 3, learning_rate = 0.02, subsample = 1.0, colsample_bytree = 1.0, L2 regularization with reg_lambda = 1.0, base_score = 0.5, eval_metric = "logloss”, and trees built with the exact algorithm (tree_method = "exact").

$$\hat{p}\left(x\right)=P\left(Y=1|x,M_{f}\right)\text{using}\;D_{c}^{\left(f,train/validation\right)}$$

*Computing admission BP contribution to predicted outcome at individual-level:* Next, we applied the trained XGBoost model to all patients in the treatment-arm and used the TreeSHAP function to compute each feature's contributions to predicted risk of poor outcome at the individual-patient level. Specifically, for each treated subject $i\in D_{T}$, the contribution of feature *j* to the predicted outcome was computed using XGBoost's native TreeSHAP implementation:

$$\emptyset_{ij}^{f}={pred_{contrib}}_{M_{f}}{\left(x_{i}\right)}_{j}$$

where $\emptyset_{ij}$ is the SHAP value attributable to feature *j* for patient *i*, and *M*_*f*_ is the fitted model. Each $\emptyset_{ij}$is a signed quantity: positive values indicate that the feature increases the predicted probability of poor outcome, whereas negative values indicate a risk-reducing contribution, relative to the model's baseline expected prediction. We specifically calculated the SHAP values for pre-randomization systolic and diastolic BP contributions to predicted poor outcome risk. Then, to identify treated patients whose predicted outcomes were most strongly influenced by admission BP, we ranked all patients in the treatment-arm by the absolute SHAP value of systolic and diastolic BP variables; where, for filter feature j∈{SBP,DBP}, patients were ranked in descending order of ${|\emptyset }_{ij}^{f}|$, and the top-*k* patients (10–50%) with the greatest systolic and/or diastolic BP variable influence on outcome were ranked:

$$C_{f}\left(q_{expl}\right)=\underset{j\in \left\{SBP,DBP\right\}}{⋃}{Top}_{k}\left({|\emptyset }_{ij}^{f}|\right)$$

*Counterfactual feature perturbation to identify subjects with a predicted risk reduction under simulated BP lowering*: Counterfactual feature perturbation mirrors the clinical reasoning process at the bedside [14], where clinicians draw on their knowledge to predict how modifying a specific risk factor – such as BP reduction – may influence an individual patient's outcome. For each candidate patient $i\in C_{f}\left(q_{expl}\right)$ from treatment group, a counterfactual feature vector $x_{i}^{CF}$ was constructed by lowering both systolic and diastolic BP by 1-to-3 standard deviation (SD) of distribution from training subcohort (listed in Supplementary Table 1). The observed predicted risk for each patient was obtained by applying the fitted model to their original feature vector:

$${\hat{p}}_{0}\left(i\right)=\hat{p}\left(x_{i}\right)$$

The counterfactual predicted risk was then estimated by applying the same model to the perturbed feature vector: ${\hat{p}}_{CF}\left(i\right)=\hat{p}\left(x_{i}^{CF}\right)$

The individual patient level risk reduction of poor outcome attributable to simulated BP lowering was defined as: $\Delta p\left(i\right)={\hat{p}}_{0}\left(i\right)-{\hat{p}}_{CF}\left(i\right)$

where positive Δp(i) indicate that the counterfactual BP reduction was associated with a decrease in predicted poor outcome risk for that individual patient. Using Δp(i) values, all patients in the intensive BP reduction treatment-arm were then ranked based on their predicted risk reduction (from 10% to 30%). Treatment-arm patients with the largest simulated reduction in predicted risk of poor outcome were considered as potential responders to intensive BP reduction.

*Exploratory, hypothesis-generating, grid search strategy for identifying treatment-arm patient subgroups with significant association of intensive BP reduction with outcome compared to held-out controls:* To identify optimal selection strategies for detecting treatment-arm subgroups most likely to benefit, we conducted a structured grid search. Patient subgroups were defined based on [1]: the magnitude of systolic/diastolic BP contributions to outcome prediction (top 10%–50% by SHAP values) [2]; the extent of predicted risk reduction (top 10%–30%) under simulated BP decreases of 1-to-3 SD; and [3] a tiered combination of SHAP- and counterfactual-based approaches (125 SHAP × counterfactual iterations). For each parameter set, we tested whether treatment independently predicted outcome using multivariable regression in a combined cohort of treatment-arm subgroup and held-out controls not used in model training. Variables showing significant associations with outcome within either treatment or control-arm were included in regression model (Supplementary Table 3). The patient selection strategy that identified a treatment-arm subgroup with the lowest statistically significant odds of poor outcome, compared to the held-out control group (Supplementary Table 4–6), was considered optimal for detecting potential treatment benefit.

*Comparison of the treatment-arm candidate responder subgroup with held-out controls meeting the same selection criteria*: We applied the optimal selection criteria to the held-out control group to approximate their use as trial enrollment criteria. The association between intensive BP reduction and outcomes was evaluated for (i) binary poor outcome and (ii) ordinal modified Rankin Scale (mRS, 0–6) score using multivariable regression models. Effect estimates are reported as odds ratios (ORs) with 95% confidence intervals (CIs). To assess the robustness of observed associations, we also performed permutation testing (5000 iterations) by randomly shuffling treatment labels within the analysis pool, defined as the combined set of selected treatment-arm patients and the held-out controls. For each iteration, outcomes were re-compared between the treatment subgroup and a control subgroup identified using the same SHAP and/or counterfactual-based selection criteria.

*Propensity score matching of treatment-arm candidate responder to held-out controls*: To enable a clinically valid comparison of outcomes, treatment-arm patients identified as likely benefiting from the intensive BP reduction were also matched to similar patients from the one-third held-out controls using 1:1 nearest-neighbor matching without replacement, based on the logit of the propensity score, and applying a caliper of 0.20 standard deviations. Covariates included in the propensity score model were selected based on their significant association with the outcome (Supplementary Table 3). The quality of the matching process was evaluated by calculating the absolute Standardized Mean Difference (SMD) for all covariates before and after matching.

*Statistics*: Continuous variables are presented as mean ± standard deviation, ordinal variables as median [interquartile range], and categorical variables as number (percentage). Group comparisons were performed using the student's *t*-test, Wilcoxon rank-sum test, and Pearson's chi-square test, as appropriate. Model prediction performance was evaluated using receiver operating characteristic (ROC) area under the curve (AUC), reported with 95% CI. The best f1 score was used to determine the optimal threshold for XGBoost classifier. Subjects without an outcome variable were excluded from our study (11 controls and 1 from the treatment arm). The XGBoost model natively accommodates missing data; thus, no imputation was required for model training, SHAP value estimation, or counterfactual feature perturbation. Subjects with missing variables were excluded from multivariable regression and propensity score matching analyses. Independent predictors of (binary) poor outcome and (0-to-6 ordinal) 3-month mRS scores were identified using logistic and ordinal regression with backward selection based on the Akaike information criterion (AIC).

## Results

*Training and validation of the machine learning model to predict ICH outcome*: There were no significant differences in the baseline characteristics of the control-arm patients in model training (n = 326) versus held-out (n = 163) subcohorts (Supplementary Table 1). The XGBoost model prediction performance characteristics are summarized in Table 1. The model achieved a ROC AUC of 0.85 (95% confidence interval, CI: 0.81–0.89) during cross-validation, with similar performance on independent validation in the held-out control subcohort (AUC = 0.83; 95% CI: 0.77–0.89) and in treatment-arm patients (AUC = 0.84; 95% CI: 0.81–0.87).

**Table 1:** Machine learning model performance across three study subcohorts.

	(Cross validation in) Control patients used for model training (n = 326)	Control patients held-out for comparison with potential treatment responders (n = 163)	Treatment-arm patients (n = 499)
Poor outcome rate	123 (37.7%)	62 (38.0%)	197 (39.5%)
AUC	0.85 (0.81–0.89)	0.83 (0.77–0.89)	0.84 (0.81–0.87)
Sensitivity	0.84 (0.77–0.89)	0.77 (0.66–0.87)	0.85 (0.8–0.89)
Specificity	0.74 (0.68–0.8)	0.72 (0.63–0.81)	0.71 (0.65–0.75)
F1 score	0.74 (0.69–0.79)	0.7 (0.62–0.76)	0.74 (0.7–0.77)
Calibration slope	1.07	0.94	0.93

Performance of the XGBoost machine learning model from cross-validation in control-arm patients used for model training and independent validation in held-out controls and treatment-arm patients.

**Fig. 2:**
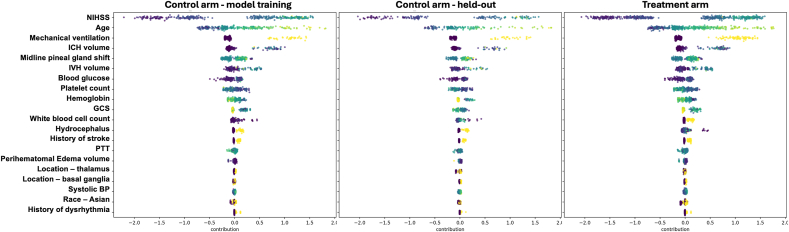
Summary (beeswarm) plot of SHAP values for top contributing features to predicted poor outcome. Each dot represents an individual patient. The x-axis (SHAP value) reflects the direction and magnitude of a feature's contribution to the predicted probability of poor outcome: positive values increase risk, whereas negative values decrease risk. Dot color encodes the standardized feature value (low: purple/blue; high: yellow). The horizontal dispersion for each feature indicates variability and overall impact across the cohort, with greater spread reflecting stronger and more heterogeneous effects. Features are ordered by the mean absolute SHAP value across all patients.

**Fig. 3:**
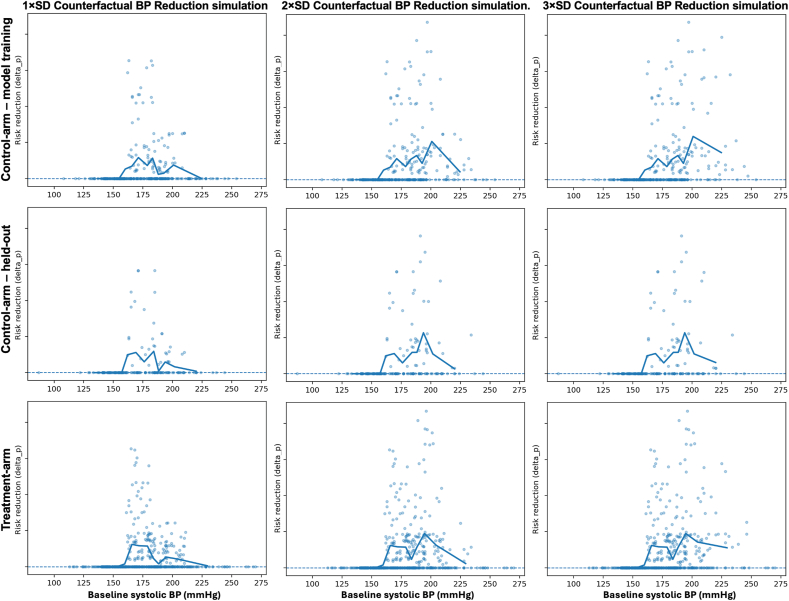
Counterfactual simulations of pre-randomization systolic BP reduction by 1–3 standard deviations (SD) relative to training subset in all study subcohorts. Each point represents the predicted change in poor outcome probability (delta_p) for one individual under simulated systolic BP reductions; and the solid line represents the locally smoothed mean risk reduction. Predicted risk reductions were heterogeneous and predominantly near-zero across all subcohorts, with potential benefit concentrated among individuals with baseline systolic BP > 160 mmHg. Larger simulated BP reductions yielded incrementally greater effects that were non-proportional and subject to diminishing outcome benefits.

**Fig. 4:**
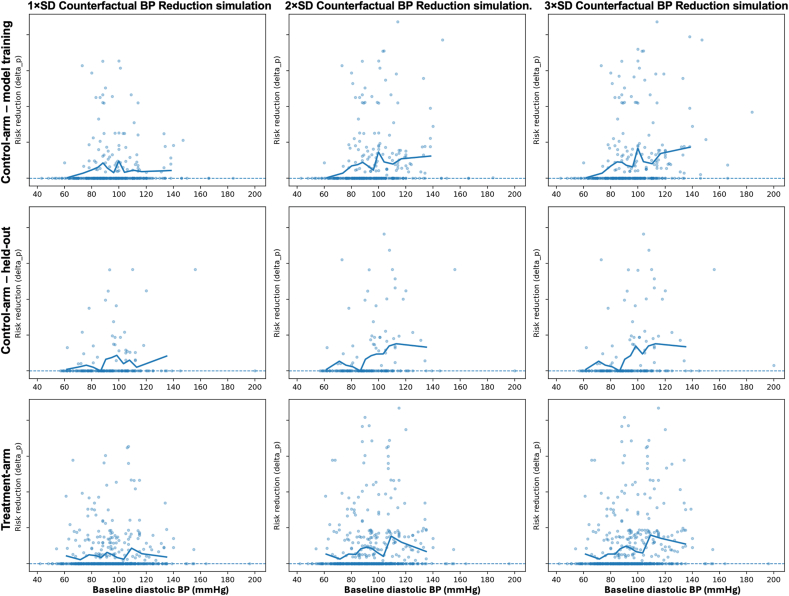
Counterfactual simulations of pre-randomization diastolic BP reduction by 1–3 standard deviations (SD) relative to training subset in all study subcohorts. Similar to systolic BP in Fig. 3, predicted risk reductions were heterogeneous and predominantly near-zero across all subcohorts, with potential benefit concentrated among individuals with diastolic BP > 80 mmHg. Larger simulated BP reductions yielded incrementally greater effects that were non-proportional and subject to diminishing outcome benefits.

*BP contribution and simulated reduction effects on predicted poor outcome*: SHAP analysis showed that NIHSS and patient age contributed most to the individual-level prediction of poor outcomes (Fig. 2). In contrast, systolic BP contributed modestly to the model, while diastolic BP was not among the top 20 predictors. The association between diastolic BP and outcome is likely mediated through its correlation with systolic BP. Counterfactual feature perturbation simulations of BP reductions for 1-to-3 standard deviations (SD) relative to the distribution in control-arm training subset (SD of 24.1 mmHg for systolic and 20.2 mmHg for diastolic BP), demonstrated modest, heterogeneous, and predominantly near-zero reductions in predicted risk of poor outcome (Fig. 3, Fig. 4), with risk reduction concentrated among individuals with systolic BP > 160 mmHg and diastolic BP > 80 mmHg. Although larger BP reductions produced incrementally greater risk-probability-changes, gains were non-proportional and varied across individuals, consistent with a nonlinear, threshold-dependent relationship between BP reduction and predicted poor outcome risk.

*Identifying treatment-arm candidate responder subgroup*: When compared with held-out controls in multivariable models adjusted for variables significantly associated with outcome (Supplementary Table 3), treatment-arm subgroups selected based on either SHAP values (top 10–50%) or predicted risk reduction from simulated BP lowering (top 10–30% under 1–3 SD reductions) failed to detect any significant association with outcome (Supplementary Tables 4 and 5). However, combining these selection strategies revealed a significant reduction in poor outcome associated with intensive BP reduction, particularly among treatment-arm patients in top 40–50% SHAP values and predicted risk reduction under 1–1.5 SD lowering of BP (Supplementary Table 6). We selected the treatment-arm subgroup (n = 56) where BP reduction was associated with the lowest significant odds ratio (OR) of poor outcome compared to held-out controls. Comparing this treatment-arm subgroup with held-out controls in multivariable analysis (Table 2), intensive BP reduction was independently associated with lower rates of poor outcome (OR = 0.21, 95% CI: 0.08–0.54, p = 0.002) and lower ordinal mRS scores (OR = 0.54, 95% CI: 0.29–0.9, p = 0.044).

**Table 2:** Independent predictors of (A) poor outcome (as binary variable) and (B) the 3-month mRS (as an ordinal variable) in the combined treatment-arm candidate responder subgroup (n = 58) and held-out controls not used in model training (n = 163) using multivariable regression.

A. Predictors of poor outcome (binary)	OR (95% CI)	P value
Intensive BP reduction therapy	0.21 (0.08–0.54)	0.002
Age	1.58 (1.05–2.43)	0.030
NIHSS	1.99 (1.33–3.16)	0.002
Sex – male	0.3 (0.13–0.67)	0.004
Race – Black	3.89 (1.34–11.7)	0.013
INR	1.42 (0.98–2.12)	0.069
ICH volume	2.01 (1.22–3.48)	0.009
Pineal gland midline shift	1.65 (1.13–2.46)	0.012
Hydrocephalus	3.84 (1.27–12.21)	0.019
Location – Thalamic	3.49 (1.44–8.78)	0.006

**B. Predictors of 3-month mRS (full range of the ordinal variable)**	**OR (95% CI)**	**P value**

Intensive BP reduction therapy	0.54 (0.29–0.9)	0.044
Age	1.72 (1.31–2.24)	0.000
NIHSS	4.57 (2.82–6.33)	0.000
GCS	1.8 (1.14–2.34)	0.002
Sex – male	0.52 (0.29–0.84)	0.017
Race – Black	2.18 (0.93–4.47)	0.054
IVH volume	1.67 (1.26–2.12)	0.003
Pineal gland midline shift	1.42 (1.13–1.89)	0.012

Stepwise backward selection multivariable logistic (A) and ordinal (B) regression analyses adjusting for variables associated with poor outcome in univariate analysis (Supplementary Table 3). Four subjects from the treatment-arm and twenty-one subjects from controls were excluded due to missing variables.

*Comparison of treatment-arm candidate responder subgroup with control subjects meeting same selection criteria:* Applying the same SHAP-based and counterfactual BP reduction criteria used to define the treatment-arm responder subgroup, we identified 19 patients from the held-out control cohort. Baseline characteristics of these subgroups are presented in Table 3, with comparisons to the remaining treatment-arm patients and held-out controls shown in Supplementary Table 7. In multivariable logistic regression analysis of the combined cohort (Supplementary Table 8), intensive BP reduction was independently associated with lower odds of binary poor outcome (OR = 0.03, 95% CI: 0–0.19, p = 0.001) and lower mRS scores (OR = 0.14, 95% CI: 0.04–0.42, p = 0.001). Permutation testing (5000 iterations), performed by randomly shuffling treatment labels within the analysis pool, indicated that the observed treatment effect was unlikely to have occurred by chance. Conditional on subgroup selection, the probability of observing a comparable treatment effect by chance among permutations was 1.3% (null pass rate = 0.0134), supporting the robustness of the observed association.

**Table 3:** Comparison of treatment-arm candidate responder subgroup with held-out control subjects meeting same selection criteria using SHAP and counterfactual BP reduction simulation.

Variable	Treated patients predicted to benefit from therapy (n = 56)	Controls fulfilling similar selection criteria (n = 19)	P value
Age	60.41 ± 11.66	62.32 ± 11.14	0.529
NIHSS	15 [11, 18]	12 [12, 15]	0.169
GCS	14 [12, 15]	15 [14, 15]	0.210
Sex (male)	33/56 (58.9%)	11/19 (57.9%)	0.937
Race – Black	12/56 (21.4%)	3/19 (15.8%)	0.747
Race – White	10/56 (17.9%)	3/19 (15.8%)	1.000
Race – Asian	30/56 (53.6%)	9/19 (47.4%)	0.640
Ethnicity – Hispanic	3/56 (5.4%)	0/19 (0.0%)	0.567
Systolic BP (mmHG)	183.1 ± 14.55	184.5 ± 14.11	0.717
Diastolic BP (mmHG)	96.91 ± 18.53	103.8 ± 16.99	0.143
Blood glucose (mg/dL)	111 ± 35.7	118.2 ± 32.65	0.420
Platelet count (1000/μL)	223.8 ± 61.56	219 ± 83.42	0.819
INR	1.004 ± 0.1206	0.9684 ± 0.07493	0.142
PTT (seconds)	28.5 ± 5.37	25.39 ± 6.568	0.074
ICH volume (mL) – baseline	15.59 ± 10.29	13.7 ± 10.13	0.510
Perihematomal edema volume (mL) – baseline	19.27 ± 10.39	16.83 ± 9.338	0.369
IVH volume (mL) – baseline	0.7206 ± 2.682	2.066 ± 3.394	0.149
Pineal shift (mm) – baseline	1.516 ± 1.713	1.227 ± 1.25	0.438
ICH volume (mL) – 24 hours	17.1 ± 13.81	16 ± 12.38	0.759
Perihematomal edema volume (mL) – 24 hours	23.54 ± 15.04	22.01 ± 10.62	0.648
Hematoma growth ratio (24 hour/baseline)	1.10 ± 0.44	1.24 ± 0.68	0.410
Hydrocephalus– baseline	4/55 (7.3%)	5/19 (26.3%)	0.043
Location – Lobar	2/55 (3.6%)	0/19 (0.0%)	1.000
Location – Thalamic	14/55 (25.5%)	4/19 (21.1%)	1.000
Location – Basal ganglia	39/55 (70.9%)	15/19 (78.9%)	0.496
3-month poor outcomes	18/56 (32.1%)	12/19 (63.2%)	0.017
3-month mRS	3 [2, 4]	4 [3, 4]	0.017

Unadjusted P values were calculated using Student's t-test, Wilcoxon rank-sum test, or Pearson chi square test.

*Comparison of treatment-arm candidate responder subgroup with propensity score-matched subjects from held-out controls:* Applying propensity score matching, we identified 41 patients from the held-out control cohort with similar baseline characteristics. The average absolute SMD for all covariates decreased from 0.57 before matching to 0.16 after matching. Baseline characteristics of these subgroups are presented in Supplementary Table 9. In multivariable logistic regression analysis of the combined cohort (Supplementary Table 10), intensive BP reduction was independently associated with lower odds of binary poor outcome (OR = 0.2, 95% CI: 0.06–0.62, p = 0.008) and was retained in the model for ordinal mRS, where it showed a similar direction of effect without reaching statistical significance (OR = 0.52, 95% CI: 0.29–0.95, p = 0.088).

## Discussion

In this post-hoc analysis, we showed the potential of explainable machine learning approaches to identify a patient subgroup in whom intensive BP reduction is associated with a lower risk of poor outcome relative to the entire held-out control cohort, subsets of held-out controls meeting the same selection criteria, and propensity score-matched controls, despite the overall neutral results of the ATACH-2 clinical trial. We found that the contribution of BP to individual-level 3-month outcomes prediction was modest, and only a small subset of patients was predicted to benefit from counterfactual simulated BP lowering. A combined strategy integrating SHAP-based feature contributions with counterfactual simulations of BP reduction – but not either method alone – effectively identified a subgroup within the treatment-arm that exhibited improved outcomes compared with held-out controls. Although our exploratory hypothesis-generating analysis is limited by the absence of formal adjustment for multiple comparisons, permutation testing indicates that the observed findings are unlikely to be due to chance. With validation in larger, representative cohorts, such approaches may support more individualized, risk-based BP management in acute ICH and help inform future trial design.

While the treatment-arm candidate responders and the control subgroup meeting the same selection criteria had higher systolic BP than the remainder of their respective cohorts, the observed differences in outcomes are unlikely to be explained by elevated BP alone and simple systolic BP thresholds are insufficient for identifying ICH patients most likely to benefit from intensive BP reduction therapy. It is notable that SHAP value analyses showed only a modest contribution of BP to overall outcome prediction. In counterfactual simulations, we also found a broadly consistent but highly heterogeneous pattern: predicted reductions in the probability of poor outcome were generally more pronounced among patients with higher baseline systolic BP, yet there was substantial overlap between regions of benefit and no benefit across all cohorts, with no clear monotonic dose–response relationship. Importantly, larger BP reductions did not uniformly translate into greater benefit and, in some cases, showed diminishing or inconsistent effects. These findings support the concept of an optimal – rather than maximal – BP reduction range. These findings are consistent with recent latent class analyses of pooled data from the four INTERACT trials and ATACH-2, which identified distinct high-systolic BP trajectories associated with worse prognosis [15]. Another post hoc analysis of ATACH-2 reported that early systolic BP reduction in the range of 55–85 mmHg within the first 2 hours was associated with more favorable outcomes in patients with mild-to-moderate ICH [16]. We extend these observations by demonstrating that predicted risk reduction is not linear and is modulated by interactions with other clinical variables. Notably, the simulated BP reduction range of 1-to-3 SD examined in our study (equivalent to a systolic BP reduction of 24.1–72.3 mmHg) broadly overlaps with the range previously proposed to confer potential outcome benefit [16].

Notably, treatment-arm candidate responders and their matched controls had higher NIHSS scores, lower admission blood glucose levels, and a higher prevalence of basal ganglia involvement compared with the remainder of their respective cohorts. Prior post hoc analyses of the ATACH-2 trial showed that patients with moderate-to-severe ICH defined by GCS <13 or NIHSS ≥10 experienced reduced rates of hematoma expansion with intensive BP lowering, but without significant disability or mortality benefit [17]. Whereas, a meta-analysis of six randomized clinical trials reported that intensive BP reduction was associated with lower rates of unfavorable outcomes, particularly among patients with lower NIHSS scores [18]. Regarding hemorrhage location, deep ICH has been associated with lower rates of hematoma expansion among patients receiving intensive BP reduction in prior post-hoc analyses, but without any significant treatment benefit, although the direction of effect has generally favored potential benefit [19,20]. Moreover, hyperglycemia has shown a consistent and independent association with poor outcomes in both the ATACH-2 and INTERACT-2 trials [21,22]. Overall, these findings highlight the complex interplay among severity of symptoms at presentation, hemorrhage location, and metabolic factors in determining the association of intensive BP reduction and outcome. The relatively modest contribution of BP to outcome prediction, coupled with these interaction effects, underscores the limitations of relying on single BP thresholds. Instead, these results support the use of composite, individualized risk stratification approaches to guide targeted antihypertensive therapy after ICH. Such characteristics may also help inform future clinical trial enrollment strategies.

Notably, although the selected treatment-arm subgroup had higher admission ICH and peri-hematomal volume than the rest of cohort, there were no differences in absolute or relative ICH volume expansion compared to held-out controls meeting same selection criteria, so the observed benefit of BP reduction was unlikely to be mediated by limitation of hematoma expansion. This is consistent with pooled INTERACT analyses [10] showing that the benefits of ultra-early BP reduction were not associated with limitation of hematoma expansion and may explain why prior post-hoc analyses showing reduced deep ICH expansion with BP lowering in the ATACH-2 dataset did not translate into overall improved clinical outcomes [19].

Prior studies have highlighted the complexity of ICH pathophysiology and underscore the need for precision anti-hypertensive therapy. While prior post-hoc analyses of ATACH-2 and INTERACT examined individual clinical and imaging factors as potential modifiers of antihypertensive treatment effect [19,23], our approach modeled multiple coexisting determinants of ICH outcomes accounting for non-linear relationships [24]. SHAP values provide individual-level contribution of each variable to poor outcome prediction [12], and counterfactual feature perturbation provides intuitive “what-if” analyses, identifying which variables and to what extent would need to change to alter a predicted outcome for a given patient [14]. We believe this framework – readily extensible to other cerebrovascular diseases – can account for the multifactorial complexity of ICH pathophysiology and guide precision therapy. Finally, our findings serve as a proof of concept, and larger population-based datasets are needed to develop robust risk models that can accurately quantify individual risk drivers and simulate treatment scenarios to guide personalized care.

This study has several other limitations. It is a retrospective, post-hoc analysis of a clinical trial dataset, and the subgroup identification strategy relied on a data-driven grid search designed to discover treatment effect heterogeneity and should therefore be considered exploratory and hypothesis-generating rather than confirmatory. As with any post hoc subgroup discovery approach, the process is susceptible to selection and optimization bias, whereby subgroups exhibiting apparently favorable treatment effects may emerge by chance because multiple candidate definitions are evaluated. Relatedly, we did not perform a formal multiplicity adjustment across the large number of candidate subgroup searches, and the reported effect estimates may therefore be optimistic. To mitigate these concerns, we incorporated a permutation-based framework that evaluates whether the observed subgroup treatment effects exceed those expected under the null hypothesis after accounting for the data-driven search procedure itself. Nevertheless, permutation testing cannot fully eliminate the risk of overfitting or guarantee reproducibility of the identified subgroups. Although we used propensity score matching to reduce confounding, residual confounding and selection bias cannot be excluded. Importantly, our models rely on ATACH-2 trial dataset with specific inclusion criteria, which limit generalizability to broader, real-world ICH populations, particularly those with more severe presentations, larger hematoma volumes, or lower admission BP. Although SHAP analysis improves interpretability of machine learning model, feature attribution does not establish causality and may be sensitive to correlated variables and model specification. In addition, the counterfactual simulations are model-based and assume that modifying a single variable – such as BP – occurs independently of other physiological changes. Thus, these “what-if” scenarios may not fully reflect real-world biological or treatment constraints and thus should be interpreted cautiously. We used pre-randomization BP, which may not fully capture dynamic BP trajectories, variability, or treatment adherence, all of which may influence outcomes. Finally, development and external validation in independent, population-based cohorts is needed to confirm the robustness and clinical utility of this approach before it can inform decision-making.

## Conclusion

Our proof-of-concept study shows the potential of explainable machine learning to identify subgroups of patients with acute ICH in whom intensive BP reduction is associated with improved outcomes, within an otherwise neutral clinical trial. In this post hoc analysis of the ATACH-2 trial, our framework – integrating XGBoost, SHAP-based interpretability, and counterfactual simulation – revealed heterogeneous and non-linear effects of BP reduction on ICH outcomes. The association between treatment and improved outcome was not uniform, but instead concentrated within a select subgroup characterized by higher systolic BP and affected by interactions with admission NIHSS, blood glucose, ICH volume, and hemorrhage location. These findings highlight the limitations of one-size-fits-all BP targets and underscore the need for individualized, multivariable risk stratification. By capturing complex relationship among clinical risk factors, this approach provides a scalable framework for precision treatment in ICH and potentially other cerebrovascular diseases. Future work should focus on training and validating these models in large, diverse cohorts to ensure generalizability and clinical applicability.

## Data availability

The datasets analyzed during the current study are not publicly available due to confidentiality restrictions mandated by original clinical trial design but are available from the clinical trial principal investigator on reasonable request.

## Author contributions

ATT performed the statistical analysis, designed the study, and drafted the initial manuscript. AIQ, JW, JZW, DR, SBM, MRS, AM, JAK, GJF, LHS, and KNS contributed to the interpretation of results, study design, and revision of the manuscript. SP conceived the study, interpreted the results, and wrote the manuscript.

## Grant support

Dr. Payabvash is supported by NINDS R01NS140459 and K23NS118056.

## Declaration of competing interest

The authors declare the following financial interests/personal relationships which may be considered as potential competing interests:

Seyedmehdi Payabvash reports financial support was provided by National Institutes of Health. Dr. Lee H. Schwamm reports consulting activities with Medtronic, LifeImage, Genentech, Penumbra. If there are other authors, they declare that they have no known competing financial interests or personal relationships that could have appeared to influence the work reported in this paper.
