# Supplementary material for: Identifying treatment-responsive patient subgroups in a neutral clinical trial of Intensive blood pressure reduction in acute intracerebral hemorrhage: A post hoc explainable machine learning analysis
Source: Neurotherapeutics. 2026 Jul 23;23(5):e00972. doi: 10.1016/j.neurot.2026.e00972 (PMC13427444; doi:10.1016/j.neurot.2026.e00972)
Supplement: Multimedia component 1 [file mmc1.docx]

**Supplemental Materials**

[**Supplementary Table 1. Baseline characteristics of control-arm patients from the ATACH-2 trial, randomly split (2:1) into subcohorts for model training and held-out comparison with treatment-arm candidate responder subgroup.** 2](#_Toc233227497)

[**Supplementary Table 2. List of variables used for training of outcome prediction model.** 3](#_Toc233227498)

[**Supplementary Table 3. The relationship of admission characteristics with poor outcome in control and treatment-arms of the ATACH-2 trial.** 4](#_Toc233227499)

[**Supplementary Table 4. Association between intensive BP reduction and poor outcome in treatment-arm subgroups selected based on SHAP contribution of admission BP to predicted poor outcome (potential treatment responders) compared with held-out controls.** 5](#_Toc233227500)

[**Supplementary Table 5. Association between intensive BP reduction and poor outcome in treatment-arm subgroups selected by simulated counterfactual BP reduction (potential treatment responders) compared with held-out controls.** 6](#_Toc233227501)

[**Supplementary Table 6. Association between intensive BP reduction therapy and poor outcome in treatment-arm subgroups selected using combined SHAP values and simulated counterfactual BP reduction (potential treatment responders) compared with held-out controls.** 7](#_Toc233227502)

[**Supplementary Table 7. Comparison of treatment-arm candidate responder subgroup and held-out controls meeting same selection criteria versus the rest of treatment-arm patients and held-out control cohort**. 12](#_Toc233227503)

[**Supplementary Table 8. Independent predictors of (A) poor outcome (as binary variable) and (B) the 3-month mRS (as an ordinal variable) in the combined cohort of treatment-arm candidate responder subgroup (n=56) and held-out controls meeting same selection criteria (n=19) using multivariable regression.** 14](#_Toc233227504)

[**Supplementary Table 9. Comparison of treatment-arm candidate responder subgroup with propensity-score-matched held-out control subjects** 15](#_Toc233227505)

[**Supplementary Table 10. Independent predictors of (A) poor outcome (as binary variable) and (B) the 3-month mRS (as an ordinal variable) in the combined cohort of treatment-arm candidate responder subgroup (n=56) and propensity-score-matched controls from held-out cohort (n=41) using multivariable regression** 16](#_Toc233227506)

# **Supplementary Table 1. Baseline characteristics of control-arm patients from the ATACH-2 trial, randomly split (2:1) into subcohorts for model training and held-out comparison with treatment-arm candidate responder subgroup.**

| **Variable** | **Control patients used for model training (n=326)** | **Control patients held out for comparison with potential treatment responders (n=163)** | **P value** |
| --- | --- | --- | --- |
| Age (years) | 61.64 ± 13.14 | 62.71 ± 12.63 | 0.388 |
| NIH Stroke Scale (NIHSS) | 11 [7, 16] | 10 [6, 14.25] | 0.260 |
| Glasgow Coma Scale (GCS) | 15 [13, 15] | 15 [14, 15] | 0.555 |
| Sex (male) | 202/326 (62.0%) | 106/163 (65.0%) | 0.508 |
| Race – Black | 40/326 (12.3%) | 15/163 (9.2%) | 0.312 |
| Race – White | 85/326 (26.1%) | 55/163 (33.7%) | 0.077 |
| Race – Asian | 185/326 (56.7%) | 85/163 (52.1%) | 0.335 |
| Ethnicity – Hispanic | 31/326 (9.5%) | 9/163 (5.5%) | 0.129 |
| Systolic blood pressure (BP, mmHG) | 175.1 ± 24.1 | 173.4 ± 24.9 | 0.465 |
| Diastolic BP (mmHG) | 94.99 ± 20.2 | 92.91 ± 20.5 | 0.288 |
| Blood glucose (mg/dL) | 137.1 ± 51.66 | 145.1 ± 73.17 | 0.218 |
| Platelet count (1000/µL) | 220.3 ± 66.7 | 221.8 ± 59.3 | 0.802 |
| International Normalized Ratio (INR) | 1.00 ± 0.16 | 0.98 ± 0.09 | 0.060 |
| Partial Thromboplastin Time (PTT, sec) | 27.65 ± 5.31 | 27 ± 6.137 | 0.258 |
| ICH volume (mL) | 13 ± 12.87 | 13.26 ± 12.84 | 0.836 |
| Perihematomal edema volume (mL) | 16.81 ± 21.63 | 16.19 ± 12.52 | 0.701 |
| Intraventricular Hemorrhage (IVH) volume (mL) | 2.46 ± 6.48 | 2.93 ± 7.58 | 0.517 |
| Pineal gland midline shift (mm) | 1.056 ± 1.771 | 0.9427 ± 1.504 | 0.467 |
| Hydrocephalus | 40/319 (12.5%) | 24/159 (15.1%) | 0.440 |
| Location – Lobar | 43/322 (13.4%) | 21/160 (13.1%) | 0.944 |
| Location – Thalamic | 96/322 (29.8%) | 48/160 (30.0%) | 0.966 |
| Location – Basal ganglia | 181/322 (56.2%) | 91/160 (56.9%) | 0.890 |
| 3-month poor outcomes | 123/326 (37.7%) | 62/163 (38.0%) | 0.947 |
| 3-month modified Rankin Scale (mRS) | 3 [1, 4] | 3 [1, 4] | 0.851 |

Values are presented as mean ± standard deviation, median [interquartile range], or n (%), as appropriate. Unadjusted P values were calculated using Student’s t-test, Wilcoxon rank-sum test, or Pearson chi square test.

# **Supplementary Table 2. List of variables used for training of outcome prediction model.**

| Biological sex of the patient |  | History of myocardial infarction |
| --- | --- | --- |
| Age at enrollment (years) |  | History of coronary artery bypass graft |
| Baseline Glasgow Coma Scale (GCS) score |  | History of hypertension |
| Baseline NIH Stroke Scale (NIHSS) score |  | History of peripheral vascular disease |
| Hispanic ethnicity indicator |  | History of hyperlipidemia |
| Black race indicator |  | History of cardiac dysrhythmia |
| White race indicator |  | History of type 1 diabetes mellitus |
| Asian race indicator |  | History of type 2 diabetes mellitus |
| Systolic blood pressure (BP) – pre-randomization |  | History of smoking |
| Diastolic BP – pre-randomization |  | History of cocaine use |
| Time to start of BP treatment |  | Intracerebral hemorrhage (ICH) location – Lobar |
| Baseline platelet count |  | ICH location – Thalamus |
| Baseline partial thromboplastin time (PTT) |  | ICH location – Basal ganglia |
| Baseline international normalized ratio (INR) |  | Infratentorial hemorrhage location |
| Baseline serum glucose level |  | Side of hemorrhage (left/right) |
| Baseline white blood cell count |  | Intraventricular hemorrhage (IVH) presence |
| Baseline hemoglobin level |  | Subarachnoid hemorrhage presence |
| Baseline hematocrit |  | Hydrocephalus presence |
| History of prior stroke |  | Pineal gland midline shift |
| History of central nervous system disease |  | ICH volume |
| History of congestive heart failure |  | IVH volume |
| History of atrial fibrillation |  | Perihematomal edema volume |

Variables used for training of explainable machine learning models to predict 3-month poor outcome (modified Rankin Scale, mRS, of 4 to 6) in acute ICH patients.

# **Supplementary Table 3. The relationship of admission characteristics with poor outcome in control and treatment-arms of the ATACH-2 trial.**

| **Variable** | **Control-arm patients (n=489)** | | | **Treatment-arm patients (n=499)** | | |
| --- | --- | --- | --- | --- | --- | --- |
|  | **Poor (n=185)** | **Favorable (n=304)** | **P value** | **Poor (n=197)** | **Favorable (n=302)** | **P value** |
| Age | 65.91 ± 13.44 | 59.62 ± 12.09 | <0.001 | 65.54 ± 13.49 | 59.71 ± 12.34 | <0.001 |
| NIHSS | 15.77 ± 7.10 | 9.07 ± 6.01 | <0.001 | 15.5 ± 5.81 | 9.29 ± 5.87 | <0.001 |
| GCS | 12.85 ± 2.60 | 14.15 ± 1.61 | <0.001 | 13 ± 2.52 | 14.16 ± 1.58 | <0.001 |
| Sex (male) | 99/185 (53.5%) | 209/304 (68.8%) | 0.001 | 114/197 (57.9%) | 189/302 (62.6%) | 0.292 |
| Race – Black | 23/185 (12.4%) | 32/304 (10.5%) | 0.518 | 38/197 (19.3%) | 35/302 (11.6%) | 0.017 |
| Race – White | 61/185 (33.0%) | 79/304 (26.0%) | 0.097 | 68/197 (34.5%) | 74/302 (24.5%) | 0.015 |
| Race – Asian | 105/185 (56.8%) | 165/304 (54.3%) | 0.593 | 106/197 (53.8%) | 179/302 (59.3%) | 0.228 |
| Ethnicity – Hispanic | 16/185 (8.6%) | 24/304 (7.9%) | 0.768 | 16/197 (8.1%) | 22/302 (7.3%) | 0.730 |
| Systolic BP (mmHG) | 171.9 ± 25.04 | 176.1 ± 23.79 | 0.067 | 176 ± 29.65 | 175.1 ± 22.71 | 0.697 |
| Diastolic BP (mmHG) | 91.77 ± 21.95 | 95.83 ± 19.07 | 0.038 | 93.09 ± 23.42 | 94.35 ± 17.56 | 0.519 |
| Blood glucose (mg/dL) | 145.9 ± 64 | 135.9 ± 56.6 | 0.086 | 139.3 ± 42.83 | 134.1 ± 50.16 | 0.226 |
| Platelet count (1000/µL) | 216.1 ± 69.66 | 223.6 ± 60.74 | 0.230 | 215.9 ± 62.48 | 224.7 ± 60.06 | 0.125 |
| INR | 0.99 ± 0.15 | 0.98 ± 0.13 | 0.316 | 1.02 ± 0.14 | 0.99 ± 0.17 | 0.022 |
| PTT (sec) | 26.92 ± 5.96 | 27.76 ± 5.35 | 0.121 | 27.04 ± 5.76 | 27.74 ± 6.3 | 0.214 |
| ICH volume (mL) | 17.91 ± 15.59 | 10.14 ± 9.75 | <0.001 | 17.54 ± 14.68 | 9.79 ± 9.06 | <0.001 |
| Perihematomal edema volume (mL) | 19.34 ± 14.51 | 14.93 ± 21.26 | <0.001 | 18.87 ± 12.66 | 13.19 ± 9.5 | <0.001 |
| IVH volume (mL) | 4.79 ± 8.27 | 1.28 ± 5.43 | <0.001 | 3.66 ± 7.01 | 0.81 ± 3.59 | <0.001 |
| Pineal shift (mm) | 1.47 ± 2.04 | 0.75 ± 1.37 | <0.001 | 1.65 ± 1.85 | 0.83 ± 1.53 | <0.001 |
| Hydrocephalus | 38/180 (21.1%) | 26/298 (8.7%) | <0.001 | 31/191 (16.2%) | 32/298 (10.7%) | 0.077 |
| Location – Lobar | 18/181 (9.9%) | 46/301 (15.3%) | 0.094 | 16/192 (8.3%) | 34/300 (11.3%) | 0.283 |
| Location – Thalamic | 63/181 (34.8%) | 81/301 (26.9%) | 0.067 | 67/192 (34.9%) | 98/300 (32.7%) | 0.609 |
| Location – Basal ganglia | 100/181 (55.2%) | 172/301 (57.1%) | 0.685 | 109/192 (56.8%) | 168/300 (56.0%) | 0.867 |

Continuous variables were compared using t-test, ordinal variables using Wilcoxon rank-sum test, and categorical variables using Pearson chi-square test. P values represent unadjusted univariable comparisons between favorable and poor outcome groups within each-arm of clinical trial.

# **Supplementary Table 4. Association between intensive BP reduction and poor outcome in treatment-arm subgroups selected based on SHAP contribution of admission BP to predicted poor outcome (potential treatment responders) compared with held-out controls.**

| **Top SHAP value for admission BP** | **Potential treatment responder (n)** | **Adjusted OR for treatment (95% CI)** | **P value** |
| --- | --- | --- | --- |
| 10% | 93 | 0.36 (0.09-1.53) | 0.167 |
| 20% | 178 | 0.52 (0.21-1.26) | 0.146 |
| 30% | 250 | 0.81 (0.4-1.64) | 0.557 |
| 40% | 315 | 0.75 (0.41-1.38) | 0.353 |
| 50% | 368 | 0.84 (0.49-1.46) | 0.545 |

SHAP values alone were used to quantify the contributions of admission systolic and diastolic BP to predicted poor outcome risk. Treatment effect is reported as adjusted odds ratio (OR) for poor outcome from multivariable regression in a combined cohort of selected treatment-arm patient subgroup (potential treatment responders) and held-out controls not used in model training. Darker purple indicates a greater number of selected patients from the treatment-arm. Lower odds of poor outcome with therapy (presumed treatment benefit) are shown in green, transitioning to yellow and then red as odds increase (across Supplementary Tables 4–6).

# **Supplementary Table 5. Association between intensive BP reduction and poor outcome in treatment-arm subgroups selected by simulated counterfactual BP reduction (potential treatment responders) compared with held-out controls.**

| **Counterfactual BP drop** | **Top poor outcome risk drop** | **Potential treatment responder (n)** | **Adjusted OR for treatment (95% CI)** | **P value** |
| --- | --- | --- | --- | --- |
| **1 SD** | 10% | 50 | 0.19 (0.02-2.21) | 0.182 |
|  | 15% | 75 | 0.34 (0.1-1.15) | 0.083 |
|  | 20% | 100 | 0.44 (0.15-1.31) | 0.140 |
|  | 25% | 125 | 0.48 (0.19-1.21) | 0.119 |
|  | 30% | 150 | 0.53 (0.22-1.27) | 0.156 |
| **1.5 SD** | 10% | 50 | 1.04 (0.08-14.05) | 0.977 |
|  | 15% | 75 | 0.51 (0.15-1.75) | 0.286 |
|  | 20% | 100 | 0.48 (0.17-1.35) | 0.167 |
|  | 25% | 125 | 0.6 (0.23-1.52) | 0.279 |
|  | 30% | 150 | 0.73 (0.32-1.7) | 0.471 |
| **2 SD** | 10% | 50 | 1.42 (0.2-10.18) | 0.725 |
|  | 15% | 75 | 0.99 (0.26-3.79) | 0.985 |
|  | 20% | 100 | 0.92 (0.35-2.42) | 0.864 |
|  | 25% | 125 | 0.87 (0.35-2.16) | 0.767 |
|  | 30% | 150 | 0.89 (0.39-2.01) | 0.770 |
| **2.5 SD** | 10% | 50 | 0.73 (0.09-5.84) | 0.768 |
|  | 15% | 75 | 0.42 (0.1-1.84) | 0.253 |
|  | 20% | 100 | 0.92 (0.36-2.38) | 0.867 |
|  | 25% | 125 | 0.79 (0.32-1.92) | 0.596 |
|  | 30% | 150 | 0.84 (0.36-1.95) | 0.680 |
| **3 SD** | 10% | 50 | 0.89 (0.1-7.63) | 0.912 |
|  | 15% | 75 | 0.69 (0.16-3.01) | 0.620 |
|  | 20% | 100 | 1.02 (0.38-2.71) | 0.965 |
|  | 25% | 125 | 0.76 (0.31-1.86) | 0.551 |
|  | 30% | 150 | 0.84 (0.36-1.97) | 0.696 |

Treatment-arm patients were selected based on top percentage of predicted risk reduction under simulated BP lowering by 1-to-3 Standard deviations (SD), derived from the control-arm training sub-cohort: 24.1 mmHg for systolic and 20.2 mmHg for diastolic BP. Treatment effect is reported as adjusted OR for poor outcome from multivariable regression in a combined cohort of selected treatment-arm patient subgroup (potential treatment responders) and held-out controls not used in model training. Darker purple indicates a greater number of selected patients from the treatment-arm. Lower odds of poor outcome with therapy (presumed treatment benefit) are shown in green, transitioning to yellow and then red as odds increase (across Supplementary Tables 4–6).

# **Supplementary Table 6. Association between intensive BP reduction therapy and poor outcome in treatment-arm subgroups selected using combined SHAP values and simulated counterfactual BP reduction (potential treatment responders) compared with held-out controls.**

| **Top SHAP value** | **Counter-factual BP drop** | **Top poor outcome risk drop** | **Potential treatment responder (n)** | **Adjusted OR for treatment (95% CI)** | **P value** | **Held-out control subjects meeting same selection criteria** | **Poor outcome OR (95% CI) associated with intensive BP reduction in treatment and control subgroups meeting selection criteria** | **P value** |
| --- | --- | --- | --- | --- | --- | --- | --- | --- |
| **10%** | **1 SD** | 10% | 10 | 0.05 (0->100) | 0.700 | 3 | 0.06 (0-1.32) | 0.206 |
|  |  | 15% | 14 | 0.04 (0->100) | 0.673 | 5 | 0.27 (0.03-2.25) | 0.211 |
|  |  | 20% | 19 | 0.12 (0->100) | 0.675 | 6 | 0.58 (0.09-3.72) | 0.566 |
|  |  | 25% | 24 | 0.18 (0->100) | 0.729 | 8 | 1 (0.19-5.22) | 1.000 |
|  |  | 30% | 28 | 0.02 (0->100) | 0.284 | 9 | 1.11 (0.23-5.43) | 0.896 |
|  | **1.5 SD** | 10% | 10 | 0.05 (0->100) | 0.700 | 3 | 0.06 (0-1.32) | 0.206 |
|  |  | 15% | 14 | 0.04 (0->100) | 0.660 | 5 | 0.18 (0.02-1.64) | 0.111 |
|  |  | 20% | 19 | 0.12 (0->100) | 0.675 | 6 | 0.58 (0.09-3.72) | 0.566 |
|  |  | 25% | 24 | 0.18 (0->100) | 0.729 | 8 | 1 (0.19-5.22) | 1.000 |
|  |  | 30% | 28 | 0.02 (0->100) | 0.284 | 9 | 1.11 (0.23-5.43) | 0.896 |
|  | **2 SD** | 10% | 10 | 0.47 (0->100) | 0.933 | 3 | 0.86 (0.05-13.48) | 0.913 |
|  |  | 15% | 14 | 0.06 (0->100) | 0.718 | 5 | 0.83 (0.1-6.78) | 0.865 |
|  |  | 20% | 19 | 0.02 (0->100) | 0.523 | 6 | 0.58 (0.09-3.72) | 0.566 |
|  |  | 25% | 24 | 0.13 (0->100) | 0.676 | 8 | 1 (0.19-5.22) | 1.000 |
|  |  | 30% | 28 | 0.01 (0-13.68) | 0.222 | 9 | 0.95 (0.19-4.68) | 0.947 |
|  | **2.5 SD** | 10% | 10 | 0.47 (0->100) | 0.933 | 3 | 0.86 (0.05-13.48) | 0.913 |
|  |  | 15% | 14 | 0.05 (0->100) | 0.696 | 5 | 0.83 (0.1-6.78) | 0.865 |
|  |  | 20% | 19 | 0.03 (0->100) | 0.586 | 6 | 0.58 (0.09-3.72) | 0.566 |
|  |  | 25% | 24 | 0.12 (0->100) | 0.657 | 8 | 1.19 (0.23-6.17) | 0.835 |
|  |  | 30% | 28 | 0.17 (0->100) | 0.690 | 9 | 1.11 (0.23-5.43) | 0.896 |
|  | **3 SD** | 10% | 10 | 0.47 (0->100) | 0.933 | 3 | 0.86 (0.05-13.48) | 0.913 |
|  |  | 15% | 14 | 0.04 (0->100) | 0.659 | 5 | 0.83 (0.1-6.78) | 0.865 |
|  |  | 20% | 19 | 0.04 (0->100) | 0.624 | 6 | 0.46 (0.07-2.99) | 0.412 |
|  |  | 25% | 24 | 0.12 (0->100) | 0.657 | 8 | 1.19 (0.23-6.17) | 0.835 |
|  |  | 30% | 28 | 0.17 (0->100) | 0.690 | 9 | 1.11 (0.23-5.43) | 0.896 |
| **20%** | **1 SD** | 10% | 18 | 0.03 (0->100) | 0.613 | 6 | 0.13 (0-1.1) | 0.147 |
|  |  | 15% | 27 | 0.01 (0->100) | 0.351 | 9 | 0.18 (0.04-0.93) | **0.032** |
|  |  | 20% | 36 | 0.01 (0-1.82) | 0.082 | 12 | 0.29 (0.07-1.13) | 0.067 |
|  |  | 25% | 45 | 0.45 (0.03-7.89) | 0.589 | 15 | 0.61 (0.18-2.06) | 0.423 |
|  |  | 30% | 54 | 0.47 (0.03-6.6) | 0.576 | 18 | 0.66 (0.22-2.01) | 0.466 |
|  | **1.5 SD** | 10% | 18 | 0.05 (0->100) | 0.598 | 6 | 0.14 (0-1.09) | **0.134** |
|  |  | 15% | 27 | 0.09 (0-15.72) | 0.358 | 9 | 0.11 (0.02-0.62) | **0.007** |
|  |  | 20% | 36 | 0.02 (0-0.76) | **0.035** | 12 | 0.24 (0.06-0.94) | **0.034** |
|  |  | 25% | 45 | 0.25 (0.03-2.23) | 0.213 | 15 | 0.46 (0.14-1.54) | 0.206 |
|  |  | 30% | 54 | 0.7 (0.11-4.48) | 0.706 | 18 | 0.53 (0.18-1.58) | 0.248 |
|  | **2 SD** | 10% | 18 | 0.01 (0->100) | 0.300 | 6 | 0.38 (0.06-2.58) | 0.317 |
|  |  | 15% | 27 | 0.01 (0-15.4) | 0.231 | 9 | 0.34 (0.07-1.59) | 0.161 |
|  |  | 20% | 36 | 0.33 (0.02-7.12) | 0.481 | 12 | 0.38 (0.1-1.48) | 0.157 |
|  |  | 25% | 45 | 0.5 (0.06-3.89) | 0.504 | 15 | 0.52 (0.16-1.7) | 0.274 |
|  |  | 30% | 54 | 0.65 (0.09-4.54) | 0.668 | 18 | 0.57 (0.19-1.71) | 0.317 |
|  | **2.5 SD** | 10% | 18 | 0.01 (0->100) | 0.380 | 6 | 0.38 (0.06-2.58) | 0.317 |
|  |  | 15% | 27 | 0.01 (0->100) | 0.394 | 9 | 0.53 (0.11-2.49) | 0.414 |
|  |  | 20% | 36 | 0.36 (0.02-7.4) | 0.505 | 12 | 0.62 (0.16-2.37) | 0.480 |
|  |  | 25% | 45 | 0.4 (0.06-2.78) | 0.352 | 15 | 0.52 (0.16-1.7) | 0.274 |
|  |  | 30% | 54 | 0.52 (0.08-3.28) | 0.483 | 18 | 0.85 (0.28-2.56) | 0.777 |
|  | **3 SD** | 10% | 18 | 0.05 (0->100) | 0.617 | 6 | 0.5 (0.08-3.27) | 0.465 |
|  |  | 15% | 27 | 0.02 (0-94.63) | 0.355 | 9 | 0.63 (0.13-2.91) | 0.548 |
|  |  | 20% | 36 | 0.44 (0.02-9.05) | 0.592 | 12 | 0.7 (0.18-2.68) | 0.601 |
|  |  | 25% | 45 | 0.56 (0.08-3.86) | 0.559 | 15 | 0.52 (0.16-1.7) | 0.274 |
|  |  | 30% | 54 | 0.53 (0.08-3.44) | 0.506 | 18 | 0.79 (0.26-2.37) | 0.668 |
| **30%** | **1 SD** | 10% | 25 | 0.01 (0-14.78) | 0.138 | 9 | 0.1 (0.02-0.55) | **0.004** |
|  |  | 15% | 38 | 0.08 (0-12.37) | 0.331 | 13 | 0.19 (0.05-0.74) | **0.012** |
|  |  | 20% | 50 | 0.17 (0.01-2.34) | 0.185 | 17 | 0.25 (0.08-0.78) | **0.014** |
|  |  | 25% | 63 | 0.13 (0.02-0.79) | **0.027** | 21 | 0.34 (0.12-0.97) | **0.039** |
|  |  | 30% | 75 | 0.18 (0.04-0.84) | **0.029** | 25 | 0.43 (0.17-1.11) | 0.078 |
|  | **1.5 SD** | 10% | 25 | 0.01 (0-244.92) | 0.386 | 9 | 0.1 (0.02-0.55) | **0.004** |
|  |  | 15% | 38 | 0.02 (0-0.63) | **0.026** | 13 | 0.19 (0.05-0.74) | **0.012** |
|  |  | 20% | 50 | 0.3 (0.05-1.75) | 0.181 | 17 | 0.3 (0.1-0.94) | **0.034** |
|  |  | 25% | 63 | 0.49 (0.11-2.19) | 0.347 | 21 | 0.39 (0.14-1.08) | 0.066 |
|  |  | 30% | 75 | 0.42 (0.1-1.77) | 0.238 | 25 | 0.42 (0.17-1.07) | 0.066 |
|  | **2 SD** | 10% | 25 | 0.01 (0-4.54) | 0.120 | 9 | 0.31 (0.06-1.51) | 0.138 |
|  |  | 15% | 38 | 0.39 (0.02-9.7) | 0.564 | 13 | 0.35 (0.1-1.28) | 0.105 |
|  |  | 20% | 50 | 0.15 (0.02-1.22) | 0.076 | 17 | 0.38 (0.12-1.18) | 0.088 |
|  |  | 25% | 63 | 0.67 (0.14-3.16) | 0.616 | 21 | 0.42 (0.15-1.16) | 0.090 |
|  |  | 30% | 75 | 0.66 (0.17-2.57) | 0.550 | 25 | 0.48 (0.19-1.21) | 0.116 |
|  | **2.5 SD** | 10% | 25 | 0.01 (0-68.25) | 0.280 | 9 | 0.31 (0.06-1.51) | 0.138 |
|  |  | 15% | 38 | 0.01 (0-1.43) | 0.069 | 13 | 0.35 (0.1-1.28) | 0.105 |
|  |  | 20% | 50 | 0.35 (0.04-3.32) | 0.362 | 17 | 0.53 (0.17-1.63) | 0.263 |
|  |  | 25% | 63 | 0.6 (0.13-2.86) | 0.524 | 21 | 0.59 (0.22-1.61) | 0.299 |
|  |  | 30% | 75 | 0.66 (0.17-2.61) | 0.556 | 25 | 0.6 (0.24-1.51) | 0.276 |
|  | **3 SD** | 10% | 25 | 0.12 (0->100) | 0.608 | 9 | 0.45 (0.1-2.12) | 0.307 |
|  |  | 15% | 38 | 0.01 (0-1.38) | 0.067 | 13 | 0.4 (0.11-1.43) | 0.152 |
|  |  | 20% | 50 | 0.59 (0.07-4.93) | 0.629 | 17 | 0.53 (0.17-1.63) | 0.263 |
|  |  | 25% | 63 | 0.6 (0.13-2.84) | 0.517 | 21 | 0.59 (0.22-1.61) | 0.299 |
|  |  | 30% | 75 | 0.66 (0.17-2.6) | 0.549 | 25 | 0.6 (0.24-1.51) | 0.276 |
| **40%** | **1 SD** | 10% | 32 | 0.09 (0-4.78) | 0.114 | 11 | 0.33 (0.08-1.34) | 0.113 |
|  |  | 15% | 48 | 0.09 (0-0.15) | **0.009** | 16 | 0.32 (0.1-1.03) | 0.051 |
|  |  | 20% | 63 | 0.08 (0.01-0.61) | **0.014** | 21 | 0.32 (0.12-0.9) | **0.026** |
|  |  | 25% | 79 | 0.11 (0.02-0.58) | **0.009** | 26 | 0.44 (0.18-1.08) | 0.069 |
|  |  | 30% | 95 | 0.18 (0.05-0.71) | **0.014** | 31 | 0.51 (0.22-1.17) | 0.108 |
|  | **1.5 SD** | 10% | 32 | 0.07 (0-6.92) | 0.260 | 11 | 0.19 (0.04-0.83) | **0.020** |
|  |  | 15% | 48 | 0.05 (0-0.79) | **0.034** | 16 | 0.3 (0.09-0.97) | **0.040** |
|  |  | 20% | 63 | 0.08 (0.01-0.48) | **0.006** | 21 | 0.45 (0.17-1.24) | 0.120 |
|  |  | 25% | 79 | 0.2 (0.05-0.79) | **0.023** | 26 | 0.71 (0.29-1.75) | 0.460 |
|  |  | 30% | 95 | 0.26 (0.08-0.91) | **0.035** | 31 | 0.52 (0.23-1.19) | 0.118 |
|  | **2 SD** | 10% | 32 | 0.17 (0.01-5.03) | 0.306 | 11 | 0.44 (0.11-1.76) | 0.238 |
|  |  | 15% | 48 | 0.37 (0.03-4.09) | 0.419 | 16 | 0.53 (0.16-1.7) | 0.282 |
|  |  | 20% | 63 | 0.39 (0.09-1.66) | 0.204 | 21 | 0.63 (0.23-1.72) | 0.367 |
|  |  | 25% | 79 | 0.53 (0.16-1.8) | 0.307 | 26 | 0.83 (0.34-2.06) | 0.694 |
|  |  | 30% | 95 | 0.53 (0.17-1.66) | 0.276 | 31 | 0.71 (0.31-1.61) | 0.411 |
|  | **2.5 SD** | 10% | 32 | 0 (0-2.01) | 0.072 | 11 | 0.44 (0.11-1.76) | 0.238 |
|  |  | 15% | 48 | 0.41 (0.05-3.59) | 0.420 | 16 | 0.58 (0.18-1.87) | 0.362 |
|  |  | 20% | 63 | 0.2 (0.03-1.18) | 0.076 | 21 | 0.68 (0.25-1.83) | 0.441 |
|  |  | 25% | 79 | 0.41 (0.12-1.42) | 0.159 | 26 | 0.88 (0.36-2.17) | 0.782 |
|  |  | 30% | 95 | 0.47 (0.15-1.45) | 0.189 | 31 | 0.68 (0.3-1.54) | 0.354 |
|  | **3 SD** | 10% | 32 | 0.12 (0-6.58) | 0.298 | 11 | 0.5 (0.13-2) | 0.323 |
|  |  | 15% | 48 | 0.66 (0.08-5.11) | 0.687 | 16 | 0.77 (0.24-2.43) | 0.657 |
|  |  | 20% | 63 | 0.3 (0.05-1.77) | 0.185 | 21 | 0.68 (0.25-1.83) | 0.441 |
|  |  | 25% | 79 | 0.52 (0.15-1.8) | 0.303 | 26 | 0.88 (0.36-2.17) | 0.782 |
|  |  | 30% | 95 | 0.43 (0.14-1.33) | 0.142 | 31 | 0.68 (0.3-1.54) | 0.354 |
| **50%** | **1 SD** | 10% | 37 | 0.04 (0-0.49) | **0.031** | 13 | 0.3 (0.08-1.11) | 0.065 |
|  |  | 15% | 56 | 0.02 (0-0.28) | **0.003** | 19 | 0.28 (0.09-0.82) | **0.017** |
|  |  | 20% | 74 | 0.09 (0.02-0.53) | **0.007** | 25 | 0.4 (0.16-1.01) | **0.049** |
|  |  | 25% | 92 | 0.12 (0.03-0.46) | **0.002** | 31 | 0.45 (0.2-1.04) | 0.059 |
|  |  | 30% | 111 | 0.1 (0.03-0.37) | **0.001** | 38 | 0.5 (0.24-1.06) | 0.067 |
|  | **1.5 SD** | 10% | 37 | 0.43 (0.02-10.55) | 0.604 | 13 | 0.41 (0.11-1.49) | 0.171 |
|  |  | 15% | 56 | 0.1 (0.01-0.73) | **0.023** | 19 | 0.4 (0.14-1.17) | 0.090 |
|  |  | 20% | 74 | 0.16 (0.04-0.66) | **0.011** | 25 | 0.56 (0.23-1.4) | 0.214 |
|  |  | 25% | 92 | 0.22 (0.06-0.76) | **0.017** | 31 | 0.58 (0.26-1.32) | 0.190 |
|  |  | 30% | 111 | 0.25 (0.08-0.78) | **0.017** | 38 | 0.63 (0.3-1.33) | 0.224 |
|  | **2 SD** | 10% | 37 | 0.37 (0.03-4.57) | 0.440 | 13 | 0.58 (0.16-2.09) | 0.406 |
|  |  | 15% | 56 | 0.24 (0.04-1.56) | 0.135 | 19 | 0.5 (0.17-1.43) | 0.193 |
|  |  | 20% | 74 | 0.41 (0.12-1.42) | 0.158 | 25 | 0.67 (0.27-1.65) | 0.379 |
|  |  | 25% | 92 | 0.46 (0.15-1.43) | 0.180 | 31 | 0.75 (0.33-1.7) | 0.491 |
|  |  | 30% | 111 | 0.39 (0.14-1.11) | 0.078 | 38 | 0.73 (0.35-1.54) | 0.412 |
|  | **2.5 SD** | 10% | 37 | 0.14 (0.01-3.76) | 0.240 | 13 | 0.71 (0.2-2.55) | 0.599 |
|  |  | 15% | 56 | 0.46 (0.07-2.95) | 0.412 | 19 | 0.62 (0.22-1.77) | 0.367 |
|  |  | 20% | 74 | 0.42 (0.12-1.44) | 0.167 | 25 | 0.74 (0.3-1.84) | 0.514 |
|  |  | 25% | 92 | 0.32 (0.1-1.01) | 0.051 | 31 | 0.75 (0.33-1.7) | 0.491 |
|  |  | 30% | 111 | 0.42 (0.15-1.19) | 0.101 | 38 | 0.76 (0.36-1.59) | 0.470 |
|  | **3 SD** | 10% | 37 | 0.41 (0.02-7.03) | 0.539 | 13 | 0.89 (0.25-3.16) | 0.856 |
|  |  | 15% | 56 | 0.8 (0.13-5.03) | 0.815 | 19 | 0.77 (0.27-2.2) | 0.632 |
|  |  | 20% | 74 | 0.6 (0.16-2.2) | 0.437 | 25 | 0.83 (0.33-2.05) | 0.679 |
|  |  | 25% | 92 | 0.43 (0.14-1.3) | 0.134 | 31 | 0.86 (0.38-1.94) | 0.712 |
|  |  | 30% | 111 | 0.48 (0.17-1.37) | 0.170 | 38 | 0.73 (0.35-1.54) | 0.412 |

Treatment-arm patients were selected based on both BP-related SHAP contributions to outcome and predicted risk reduction under simulated BP lowering. Treatment effect is reported as adjusted OR for poor outcome from multivariable regression in a combined cohort of selected treatment-arm patient subgroup (potential treatment responders) and held-out controls not used in model training. Darker purple indicates a greater number of selected patients from the treatment-arm. Lower odds of poor outcome with therapy (presumed treatment benefit) are shown in green, transitioning to yellow and then red as odds increase (across Supplementary Tables 4–6). The selected iteration of SHAP and counterfactual BP reduction is highlighted with pink.

# **Supplementary Table 7. Comparison of treatment-arm candidate responder subgroup and held-out controls meeting same selection criteria versus the rest of treatment-arm patients and held-out control cohort**.

| **Variable** | **Treatment-arm patients (n=499)** | | | **Control-arm held-out not used in training (n=163)** | | |
| --- | --- | --- | --- | --- | --- | --- |
|  | **Unlikely to respond (n=443)** | **Potential responder (n=56)** | **P value** | **Controls fulfilling same selection criteria (n=19)** | **Controls not fulfilling same selection criteria (n=144)** | **P value** |
| Age | 62.22 ± 13.28 | 60.41 ± 11.66 | 0.286 | 62.32 ± 11.14 | 62.76 ± 12.85 | 0.875 |
| NIHSS | 10 [6, 16] | 15 [11, 18] | <0.001 | 12 [12, 15] | 9 [6, 14] | 0.006 |
| GCS | 15 [13, 15] | 14 [12, 15] | 0.021 | 15 [14, 15] | 15 [13, 15] | 0.505 |
| Sex (male) | 270/443 (60.9%) | 33/56 (58.9%) | 0.771 | 11/19 (57.9%) | 95/144 (66.0%) | 0.488 |
| Race – Black | 61/443 (13.8%) | 12/56 (21.4%) | 0.127 | 3/19 (15.8%) | 12/144 (8.3%) | 0.388 |
| Race – White | 132/443 (29.8%) | 10/56 (17.9%) | 0.062 | 3/19 (15.8%) | 52/144 (36.1%) | 0.078 |
| Race – Asian | 255/443 (57.6%) | 30/56 (53.6%) | 0.570 | 9/19 (47.4%) | 76/144 (52.8%) | 0.657 |
| Ethnicity – Hispanic | 35/443 (7.9%) | 3/56 (5.4%) | 0.788 | 0/19 (0.0%) | 9/144 (6.2%) | 0.600 |
| Systolic BP (mmHG) | 174.5 ± 26.58 | 183.1 ± 14.55 | <0.001 | 184.5 ± 14.11 | 171.9 ± 25.62 | 0.003 |
| Diastolic BP (mmHG) | 93.47 ± 20.24 | 96.91 ± 18.53 | 0.199 | 103.8 ± 16.99 | 91.47 ± 20.54 | 0.007 |
| Blood glucose (mg/dL) | 139.4 ± 47.87 | 111 ± 35.7 | <0.001 | 118.2 ± 32.65 | 148.8 ± 76.41 | 0.003 |
| Platelet count (1000/µL) | 221 ± 61.1 | 223.8 ± 61.56 | 0.744 | 219 ± 83.42 | 222.2 ± 55.6 | 0.873 |
| International Normalized Ratio (INR) | 1.00 ± 0.1634 | 1.00 ± 0.12 | 0.833 | 0.97 ± 0.07 | 0.98 ± 0.10 | 0.682 |
| Partial Thromboplastin Time (PTT, sec) | 27.33 ± 6.182 | 28.5 ± 5.37 | 0.137 | 25.39 ± 6.57 | 27.22 ± 6.07 | 0.263 |
| ICH volume (mL) – baseline | 12.42 ± 12.33 | 15.59 ± 10.29 | 0.043 | 13.7 ± 10.13 | 13.21 ± 13.17 | 0.858 |
| Perihematomal edema volume (mL) – baseline | 14.88 ± 11.18 | 19.27 ± 10.39 | 0.005 | 16.83 ± 9.34 | 16.11 ± 12.89 | 0.779 |
| IVH volume (mL) – baseline | 2.06 ± 5.59 | 0.72 ± 2.68 | 0.004 | 2.07 ± 3.397 | 3.034 ± 7.95 | 0.372 |
| Pineal shift (mm) | 1.104 ± 1.7 | 1.516 ± 1.713 | 0.098 | 1.23 ± 1.25 | 0.90 ± 1.54 | 0.314 |
| ICH volume (mL) – 24 hours | 14.8 ± 16.12 | 17.1 ± 13.81 | 0.272 | 16 ± 12.38 | 15.75 ± 16.33 | 0.942 |
| Perihematomal edema volume (mL) – 24 hours | 20.44 ± 16.57 | 23.54 ± 15.04 | 0.171 | 22.01 ± 10.62 | 19.9 ± 13.95 | 0.465 |
| Hydrocephalus | 59/434 (13.6%) | 4/55 (7.3%) | 0.187 | 5/19 (26.3%) | 19/140 (13.6%) | 0.170 |
| Location – Lobar | 48/437 (11.0%) | 2/55 (3.6%) | 0.089 | 0/19 (0.0%) | 21/141 (14.9%) | 0.080 |
| Location – Thalamic | 151/437 (34.6%) | 14/55 (25.5%) | 0.178 | 4/19 (21.1%) | 44/141 (31.2%) | 0.365 |
| Location – Basal ganglia | 238/437 (54.5%) | 39/55 (70.9%) | 0.020 | 15/19 (78.9%) | 76/141 (53.9%) | 0.039 |
| 3-month poor outcomes | 179/443 (40.4%) | 18/56 (32.1%) | 0.233 | 12/19 (63.2%) | 50/144 (34.7%) | 0.016 |
| 3-month mRS | 3 [1, 4] | 3 [2, 4] | 0.944 | 4 [3, 4] | 3 [1, 4] | 0.006 |

Values are presented as mean ± standard deviation, median [interquartile range], or n (%), as appropriate. Unadjusted P values were calculated using Student’s t-test, Wilcoxon rank-sum test, or Pearson chi square test.

# **Supplementary Table 8. Independent predictors of (A) poor outcome (as binary variable) and (B) the 3-month mRS (as an ordinal variable) in the combined cohort of treatment-arm candidate responder subgroup (n=56) and held-out controls meeting same selection criteria (n=19) using multivariable regression.**

| **A. Predictors of poor outcome (binary)** | **OR (95% CI)** | **P value** |
| --- | --- | --- |
| Intensive BP reduction therapy | 0.03 (0-0.19) | 0.001 |
| Age | 2.9 (1.32-7.66) | 0.015 |
| NIHSS | 2.95 (1.25-8.23) | 0.021 |
| INR | 2.48 (1.26-5.47) | 0.013 |
| ICH volume | 3.07 (1.14-10.08) | 0.039 |
| Pineal gland midline shift | 3.72 (1.66-11.17) | 0.006 |
| Location – Thalamic | 8.01 (0.99-81) | 0.051 |
|  |  |  |
| **B. Predictors of 3-month mRS (full range of the ordinal variable)** | **OR (95% CI)** | **P value** |
| Intensive BP reduction therapy | 0.14 (0.04-0.42) | 0.001 |
| NIHSS | 0.21 (0.07-0.55) | 0.001 |
| Sex – male | 2.22 (1.39-3.64) | 0.002 |
| INR | 1.85 (1.14-3.13) | 0.016 |
| Pineal gland midline shift | 2.14 (1.32-3.55) | 0.025 |
| Hydrocephalus | 5.52 (1.3-26.44) | 0.003 |

Four subjects from the treatment-arm and two subjects from controls were excluded due to missing variables.

# **Supplementary Table 9. Comparison of treatment-arm candidate responder subgroup with propensity-score-matched held-out control subjects**

| **Variable** | **Treated patients predicted to benefit from therapy (n=56)** | **Controls fulfilling similar selection criteria (n=41)** | **P value** |
| --- | --- | --- | --- |
| Age | 63.44 ± 11.19 | 60.41 ± 11.66 | 0.202 |
| NIHSS | 15 [10, 20] | 15 [11, 18] | 0.485 |
| GCS | 14 [12, 15] | 14 [12, 15] | 0.828 |
| Sex (male) | 28 (68.3%) | 33 (58.9%) | 0.346 |
| Race – Black | 5 (12.2%) | 12 (21.4%) | 0.237 |
| Race – White | 9 (22.0%) | 10 (17.9%) | 0.616 |
| Race – Asian | 23 (56.1%) | 30 (53.6%) | 0.805 |
| Ethnicity – Hispanic | 0 (0.0%) | 3 (5.4%) | 0.132 |
| Systolic BP (mmHG) | 185.88 ± 21.35 | 183.14 ± 14.55 | 0.455 |
| Diastolic BP (mmHG) | 97.93 ± 19.40 | 96.91 ± 18.53 | 0.794 |
| Blood glucose (mg/dL) | 119.80 ± 30.06 | 110.97 ± 35.70 | 0.202 |
| Platelet count (1000/µL) | 215.54 ± 55.75 | 223.82 ± 61.56 | 0.497 |
| INR | 1.00 ± 0.11 | 1.00 ± 0.12 | 0.726 |
| PTT (seconds) | 27.12 ± 6.64 | 28.50 ± 5.37 | 0.26 |
| ICH volume (mL) – baseline | 15.31 ± 13.33 | 15.59 ± 10.29 | 0.908 |
| Perihematomal edema volume (mL) – baseline | 19.83 ± 14.82 | 19.27 ± 10.39 | 0.829 |
| IVH volume (mL) – baseline | 1.98 ± 6.96 | 0.72 ± 2.68 | 0.229 |
| Pineal shift (mm) – baseline | 0.83 ± 1.17 | 1.52 ± 1.71 | 0.032 |
| ICH volume (mL) – 24 hours | 20.13 ± 17.37 | 17.10 ± 13.81 | 0.357 |
| Perihematomal edema volume (mL) – 24 hours | 25.17 ± 13.74 | 23.54 ± 15.04 | 0.598 |
| Hydrocephalus– baseline | 7 (17.5%) | 4 (7.3%) | 0.124 |
| Location – Lobar | 5 (12.2%) | 2 (3.6%) | 0.105 |
| Location – Thalamic | 6 (14.6%) | 15 (26.8%) | 0.151 |
| Location – Basal ganglia | 30 (73.2%) | 39 (69.6%) | 0.705 |
| 3-month poor outcomes | 17 (41.5%) | 18 (32.1%) | 0.345 |
| 3-month mRS | 3 [2, 4] | 3 [2, 4] | 0.626 |

Four subjects from the treatment-arm and twenty-one subjects from held-out controls were excluded from propensity score matching due to missing variables.

# **Supplementary Table 10. Independent predictors of (A) poor outcome (as binary variable) and (B) the 3-month mRS (as an ordinal variable) in the combined cohort of treatment-arm candidate responder subgroup (n=56) and propensity-score-matched controls from held-out cohort (n=41) using multivariable regression**

| **A. Predictors of poor outcome (binary)** | **OR (95% CI)** | **P value** |
| --- | --- | --- |
| Intensive BP reduction therapy | 0.2 (0.06-0.62) | 0.008 |
| NIHSS | 2.02 (1.13-3.9) | 0.022 |
| Sex (male) | 0.28 (0.08-0.89) | 0.038 |
| INR | 1.97 (1.15-3.68) | 0.020 |
| Perihematomal edema volume | 2.1 (1.03-4.63) | 0.050 |
| Pineal gland midline shift | 1.96 (1.14-3.67) | 0.023 |
| Location – Thalamic | 8.32 (2.02-40.19) | 0.005 |
|  |  |  |
| **B. Predictors of 3-month mRS (full range of the ordinal variable)** | **OR (95% CI)** | **P value** |
| Intensive BP reduction therapy | 0.52 (0.29-0.95) | 0.088 |
| NIHSS | 2.57 (1.88-3.58) | <0.001 |
| Sex (male) | 0.37 (0.21-0.65) | 0.002 |
| INR | 1.48 (1.13-1.95) | 0.003 |
| IVH volume | 1.46 (1.05-2.07) | 0.009 |
| Hydrocephalus– baseline | 3.29 (1.47-7.53) | 0.002 |
| Pineal gland midline shift | 1.53 (1.17-2.01) | 0.003 |

Stepwise backward selection multivariable logistic (A) and ordinal (B) regression analyses adjusting for variables associated with poor outcome in univariate analysis (Supplementary Table 3). Four subjects from the treatment-arm and twenty-one subjects from held-out controls were excluded from propensity score matching due to missing variables.
